# T-cell metabolic reprogramming in systemic lupus erythematosus: linking intracellular metabolism to effector differentiation and senescent-like/exhausted-like states

**DOI:** 10.3389/fimmu.2026.1961063

**Published:** 2026-10-12

**Authors:** Yi Zou, Zhichu Ren, Yuki Kudo, Michihito Kono

**Affiliations:** Department of Rheumatology, Endocrinology and Nephrology, Faculty of Medicine and Graduate School of Medicine, Hokkaido University, Sapporo, Japan

**Keywords:** exhausted-like T cells, immunometabolism, metabolic reprogramming, senescent-like T cells, systemic lupus erythematosus

## Abstract

Systemic lupus erythematosus (SLE) is a chronic autoimmune disease characterized by immune dysregulation and multiorgan inflammation. T-cell metabolic reprogramming has recently emerged as an active area of investigation in the pathogenesis of SLE. This metabolic reprogramming, which involves glycolysis, mitochondrial oxidative phosphorylation, glutamine metabolism, and lipid metabolism, is associated not only with functional alterations in SLE T cells but also with changes in their differentiation and proliferation, potentially contributing to disease pathogenesis and progression. Moreover, metabolic alterations, including mitochondrial dysfunction and reactive oxygen species accumulation, in the context of persistent T-cell activation may contribute to phenotypic transitions toward senescent-like or exhausted-like states. These two partially overlapping but biologically distinct cellular phenotypes may represent divergent cellular responses to chronic inflammatory stimulation. Rather than representing mutually exclusive states or a simple loss of function, both may contribute to the perpetuation of chronic inflammation, immune imbalance and disease activity. This review summarizes recent advances in T-cell metabolic reprogramming in SLE and its potential relationship to senescent-like and exhausted-like T-cell states.

## 1 Introduction

Systemic lupus erythematosus (SLE) is a highly heterogeneous autoimmune disease characterized by diverse clinical manifestations, ranging from mild skin, mucosal, and joint involvement to severe, organ-threatening manifestations, involving the renal, neuropsychiatric, hematologic, or cardiopulmonary manifestations (1, 2). The serological profile is equally complex, featuring various autoantibodies and hypocomplementemia (3). Such extensive heterogeneity is also reflected in T-cell abnormalities, including the expansion of follicular helper T (Tfh) cells, an imbalance of T helper 17 (Th17)/regulatory T (Treg) cells, or altered CD8^+^ T-cell function. Thus, T-cell abnormalities in SLE do not simply reflect a generalized increase in immune activation but rather result from the combined effects of chronic inflammatory stimulation, tissue-specific microenvironmental differences, and altered intracellular metabolic states. Moreover, SLE CD4^+^ T cells exhibit enhanced glycolysis and mitochondrial oxidative phosphorylation (OXPHOS), suggesting that cellular metabolic reprogramming may contribute to aberrant T-cell activation and disease pathogenesis (4). Based on these findings, this review discusses the features of immunometabolic reprogramming in SLE T cells.

## 2 SLE T-cell metabolic reprogramming and its impact on pathogenic T-cell states

While quiescent T cells rely on mitochondrial OXPHOS and fatty acid oxidation (FAO), they can rapidly adapt their substrate utilization upon activation to support proliferation, cytokine production, and lineage commitment. For instance, in SLE CD4^+^ T cells, the simultaneous enhancement of glycolysis meets increased energetic demand (5, 6), reshape T-cell differentiation and function (7), and may sustain pathogenic phenotypes that drive SLE progression. Consequently, metabolic reprogramming serves as a pivotal mechanism governing T-cell pathogenicity in SLE.

### 2.1 Metabolic reprogramming of glycolysis: T-cell activation, Th17/Treg imbalance and the Tfh-B cell axis

Accelerated glycolysis, representing a hallmark of dysregulated T-cell metabolism in SLE (8) and partially reflecting metabolic adaptation under aberrant T-cell receptor (TCR)/Ca^2+^ signaling, is intimately linked to the Th17/Treg imbalance. Calcium/calmodulin-dependent protein kinase IV (CaMK4) serves as a critical node bridging aberrant TCR/Ca^2+^ signaling and this metabolic reprogramming. Altered calcium fluxes stimulate CaMK4, which upregulates glucose transporter type 1 through the protein kinase B (AKT)/mechanistic target of rapamycin (mTOR) to enhance glycolysis, while concurrently driving cAMP responsive element modulator α-mediated interleukin (IL)-21 transcription, collectively promoting Th17 differentiation (9–11). Additionally, CaMK4 activates pyruvate kinase M2 (PKM2) to drive glycolysis in T helper 1 (Th1)/Th17 cells in the experimental autoimmune encephalomyelitis (EAE) mouse model (12), it conversely regulates the platelet-phosphofructokinase (PFKP) to shift Treg metabolism from OXPHOS to glycolysis, thereby impairing their functional stability in a lupus-prone B6.*lpr* mice with supporting evidence from CD4^+^ T cells of patients with SLE (**Figure 1**) (13). Thus, CaMK4 functions as a metabolic hub, exploiting PKM2 and PFKP as distinct glycolytic switches to fuel inflammatory Th17 differentiation and exacerbate Treg dysfunction.

**Figure 1:**
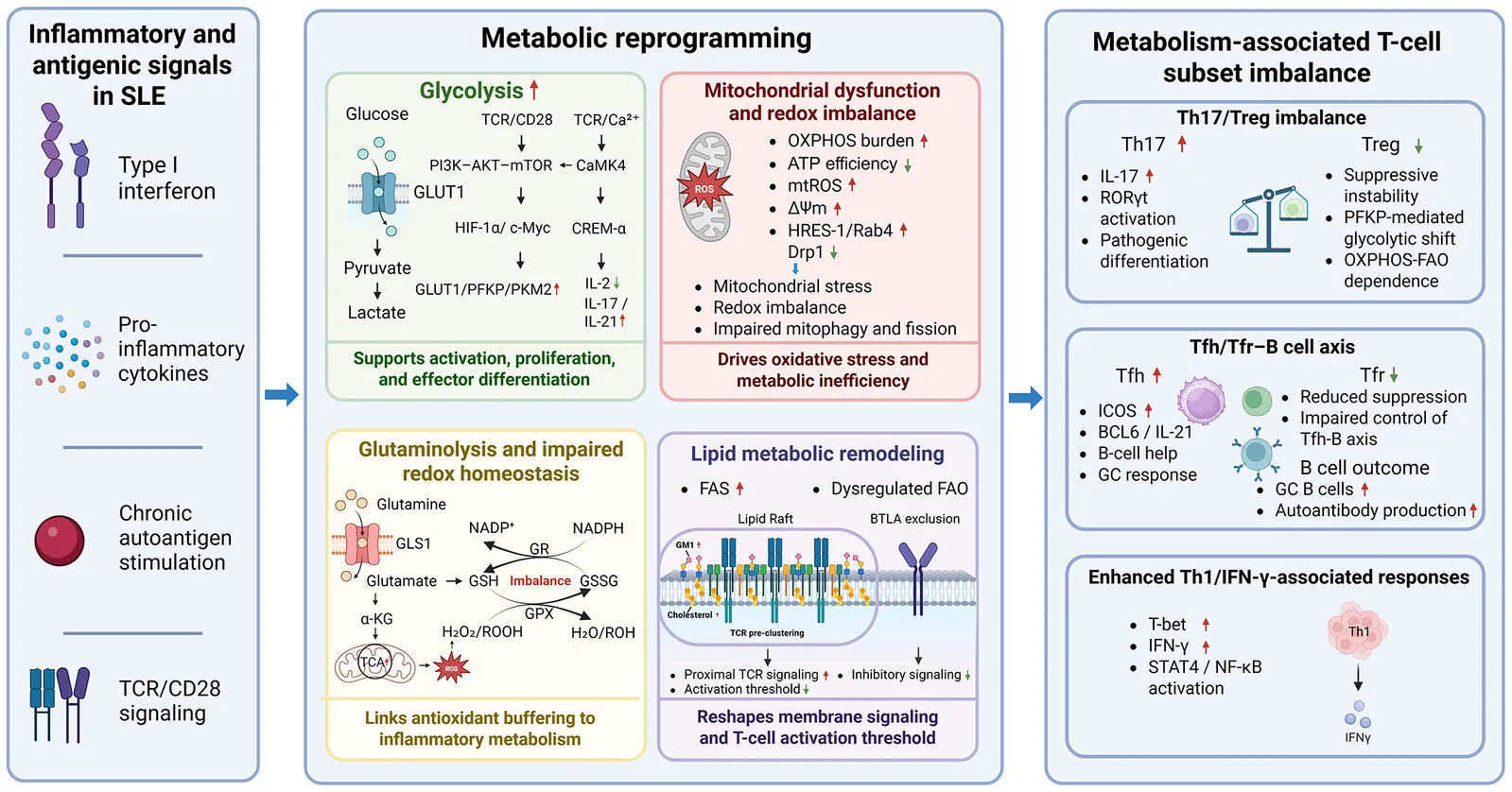
Metabolic reprogramming and T-cell subset imbalance in SLE. In SLE, type I interferons, pro-inflammatory cytokines, chronic autoantigen stimulation, and TCR/CD28 costimulation reshape T-cell metabolism. Enhanced glycolysis, mitochondrial dysfunction, impaired redox homeostasis, and lipid metabolic remodeling alter T-cell activation and effector differentiation. Collectively, these changes promote Th17/Treg imbalance, enhanced Tfh–B-cell responses with reduced Tfr-mediated restraint, increased germinal-center activity and autoantibody production, and augmented Th1/IFN-γ-associated inflammation. Upward and downward arrows indicate increased and decreased levels or activity, respectively. PI3K, phosphoinositide 3-kinase; AKT, protein kinase B; mTOR, mechanistic target of rapamycin; GLUT1, glucose transporter 1; HIF-1α, hypoxia-inducible factor 1α; c-Myc, MYC proto-oncogene protein; PFKP, platelet-type phosphofructokinase; PKM2, pyruvate kinase M2; CaMK4, calcium/calmodulin-dependent protein kinase IV; CREM-α, cAMP-responsive element modulator α; OXPHOS, oxidative phosphorylation; ROS, reactive oxygen species; mtROS, mitochondrial reactive oxygen species; ΔΨm, mitochondrial membrane potential; HRES-1/Rab4, human T-cell lymphotropic virus-related endogenous sequence 1-encoded Rab4 GTPase; Drp1, dynamin-related protein 1; GLS1, glutaminase 1; α-KG, α-ketoglutarate; GR, glutathione reductase; GSH, reduced glutathione; GSSG, oxidized glutathione; GPX, glutathione peroxidase; H_2_O_2_, hydrogen peroxide; ROOH, organic hydroperoxide; ROH, corresponding alcohol product; FAS, fatty acid synthesis; FAO, fatty acid oxidation; GM1, monosialotetrahexosylganglioside 1; BTLA, B- and T-lymphocyte attenuator; RORγt, retinoic acid receptor-related orphan receptor γt; ICOS, inducible T-cell costimulator; BCL6, B-cell lymphoma 6; T-bet, T-box transcription factor TBX21; STAT4, signal transducer and activator of transcription 4; NF-κB, nuclear factor κB.

Acting as a core kinase, mTOR is activated by TCR/CD28 co-stimulation through the phosphoinositide 3-kinase/AKT signaling cascade to drive glycolysis and anabolism. Downstream, mTOR enhances hypoxia-inducible factor-1α (HIF-1α) expression (14, 15), thereby amplifying glycolytic flux and transactivating retinoic acid receptor-related orphan receptor gamma t. This molecular event promotes the polarization and proliferation of inflammatory Th17 cells, while simultaneously compromising the proportion and functional stability of Tregs, exacerbating the Th17/Treg imbalance (**Figure 1**) (16, 17). Consistently, a previous study demonstrated that HIF-1α silencing ameliorated lupus manifestations in MRL/*lpr* mice (18), underscoring HIF-1α as a potential therapeutic target in future. Interestingly, multiple cellular metabolites, including lactate, succinate, and itaconate, can influence Th17 and Treg differentiation and function (19–21).

Moreover, glycolysis facilitates antibody production via the Tfh-B cell axis (22). Tfh cells rely on key modular programs, such as inducible T-cell co-stimulator (ICOS) and B-cell lymphoma 6 (BCL-6), to sustain their interaction with B cells. In SLE, aberrant Tfh-cell expansion potentiates abnormal germinal center (GC) reactions and promotes B-cell differentiation and antibody production (23–25). Additionally, Tfh cells exhibit dysregulated glycolysis that interacts reciprocally with Toll-like receptor 7 signaling (26); inhibition of glucose metabolism selectively reduces autoreactive Tfh cells, GC B cells, and anti-double-stranded DNA IgG levels in lupus-prone mouse models, indicating that pathogenic Tfh cells exert a specific reliance on glycolysis (22). Most importantly, glycolytic enzymes themselves exert non-metabolic functions. For example, dimeric PKM2 regulates Tfh-cell differentiation through BCL6-related transcriptional regulation; conversely, inducing PKM2 tetramerization successfully inhibits this differentiation and reduces inflammatory injury in lupus-prone mouse models, as the tetrameric form is retained in the cytoplasm and unable to translocate to the nucleus (27, 28).

Taken together, glycolytic metabolic reprogramming in SLE T cells operates through the CaMK4 and mTOR signaling cascades, as well as the Tfh-B cell axis, to collectively drive the Th17/Treg imbalance, autoantibody production, and pathogenic T-cell states, thereby perpetuating a self-reinforcing immunometabolic network.

### 2.2 Dysregulation of mitochondrial oxidative metabolism

Previous research has shown that dual inhibition of glycolysis and mitochondrial oxidative metabolism effectively alleviates lupus-like immune abnormalities in lupus-prone mice (8). However, Peter Gergely Jr. et al. identified a chronic aberration in mitochondrial membrane potential within SLE T cells, accompanied by diminished ATP levels and accelerated reactive oxygen species (ROS) production (29). These findings indicate a paradoxical mitochondrial phenotype in SLE T cells: increased mitochondrial activity or membrane hyperpolarization does not necessarily translate into efficient ATP production (29). Instead, this dysfunction manifests as heightened stress on the electron transport chain, exhaustion of redox buffering capacity, and severe mitochondrial stress (**Figure 1**) (30, 31). Mitochondrial metabolic reprogramming is closely linked to ROS accumulation and mitochondrial quality control (32, 33). ROS function as signaling molecules involved in T-cell differentiation and functional remodeling. Specifically, ROS accumulation promotes Th17 differentiation by increasing epidermal growth factor receptor expression and phosphorylation, thereby promoting its nuclear translocation and interaction with signal transducer and activator of transcription (STAT) 3 interaction (34). In addition, ROS-mediated signaling may contribute to STAT4 phosphorylation, induce nuclear factor kappa B nuclear translocation, and increase interferon-γ (IFN-γ) production, collectively augmenting CD4^+^ T-cell inflammatory effector functions (35). Notably, impaired mitochondrial fission and mitophagy have been reported in SLE T cells (33, 36), potentially promoting the accumulation of dysfunctional mitochondria and ROS, thereby further disrupting mitochondrial homeostasis (**Figure 1**).

### 2.3 Glutathione (GSH) homeostatic disruption and glutaminolysis

In homeostatic T cells, GSH is one of the major antioxidant molecules responsible for maintaining ROS homeostasis. It is oxidized to glutathione disulfide during ROS scavenging, and is subsequently recycled back to its reduced state via glutathione reductase and nicotinamide adenine dinucleotide phosphate (NADPH) (37). However, the confluence of aberrant ROS accumulation, chronic mitochondrial stress, and defective NADPH provisioning precipitates a severe mismatch between GSH supply and demand (**Figure 1**) (38, 39). Furthermore, glutamine is converted to glutamate by glutaminase 1 (GLS1). Glutamate can subsequently contribute to GSH synthesis for redox homeostasis and be converted to α-ketoglutarate to fuel the tricarboxylic acid cycle (**Figure 1**), thus supporting both energy metabolism and metabolically regulated signaling (40, 41). Glutaminolysis or GLS1 inhibition reduced glycolytic activity and ameliorated lupus-like disease in MRL/*lpr* mice according to various research, while also suppressing Th17 differentiation in CD4^+^ T cells from SLE patients *in vitro* (40, 42, 43). Zhang et al. reported that glutaminolysis inhibition regulated the Th17/Treg balance, and reduced the proportions of Tfh cells and activated B cells (43). More recently, autoreactive Tfh cells in lupus-prone mice have been shown to exhibit elevated glutamine metabolic demands compared to control Tfh cells. Besides, GLS1 inhibition was found to diminish the expression of ICOS and other molecules critical for Tfh cell function (44). However, the evidence supporting the role and regulation of glutamine metabolism in SLE remains limited and requires further validation.

### 2.4 Lipid metabolic remodeling: membrane signaling, fatty acid metabolism, and plasticity in T-cell function

Lipid metabolic remodeling provides another pivotal perspective for understanding aberrant T-cell activation in SLE and regulate T-cell states through distinct mechanistic axes. Activated CD4^+^ T cells utilize glycolytic acetyl-coenzyme A for *de novo* fatty acid synthesis (FAS) to support Th17 differentiation; in contrast, Tregs tend to rely on FAO and OXPHOS for functional stability (45). Iwata et al. showed that enhanced FAS is associated with Th1 differentiation and excessive IFN-γ production (46). Furthermore, an aberrant membrane lipid environment, characterized by increased monosialotetrahexosylganglioside and cholesterol levels, promotes premature TCR clustering and lipid raft expansion. This abnormal membrane reorganization not only lowers the threshold for T-cell activation but also physically excludes B and T lymphocyte attenuator (BTLA) from the immunological synapse, impairing its inhibitory effect (**Figure 1**) (5, 47). Sawaf et al. further suggested that while SLE patients exhibit impaired BTLA function, normalizing lipid metabolism can restore its recruitment and co-inhibitory capacity (47). In addition, a study utilizing an atherosclerosis-associated lipid environment in a lupus model showed that atherogenic dyslipidemia induced IL-27 production by splenic dendritic cells in a Toll-like receptor 4-dependent manner. This signaling cascade promoted CXCR3^+^ Tfh differentiation while suppressing follicular regulatory T (Tfr) cells, thereby driving excessive GC responses (48). Thus, lipid metabolism not only regulates Th17-, Th1-, Treg-cell differentiation, but also modulates T-cell function by remodeling lipid raft architecture and TCR signaling. Additionally, systemic lipid dysregulation can further influence T-cell differentiation and function, ultimately amplify GC responses and promote autoantibody production.

### 2.5 Metabolic pathway crosstalk and the pathogenic states of SLE T cells

Overall, metabolic reprogramming in SLE T cells constitutes an interconnected and context-dependent network rather than a simple shift between glycolysis and oxidative metabolism. Although simultaneous enhancement of glycolysis and OXPHOS has been observed in some SLE CD4^+^ T-cell populations, SLE T-cell metabolism is heterogenous, with some studies highlighting the coexistence of metabolic hyperactivation, impaired bioenergetic efficiency and redox control (49, 50). Metabolic dysregulation in SLE is not restricted to individual immune-cell populations but may be amplified through intercellular crosstalk. Metabolic dysregulation in SLE extends beyond T cells. ROS, mitochondrial fitness, and NAD^+^ homeostasis have been implicated in regulating plasmacytoid dendritic cells (pDCs) activation and IFN-I production (51), while pDCs from patients with SLE exhibit an increased capacity to stimulate T cells (52). Beyond transient activation, this metabolic reprogramming drives T cells toward relatively stable, pathobiological phenotypes. The persistent inflammatory milieu in SLE, coupled with recurrent antigenic stimulation, perpetuates T-cell receptor engagement and contributes to telomere shortening and reduced replicative potential (53). Crucially, the downstream consequences—excessive ROS, oxidative stress, and DNA damage—trigger T-cell transitions toward senescent-like or exhausted-like states (54, 55). The subsequent sections will delineate how metabolic reprogramming may contribute to or reinforce T-cell fate decisions and immune dysregulation in SLE, focusing specifically on these two paradigms (**Figure 2**).

**Figure 2:**
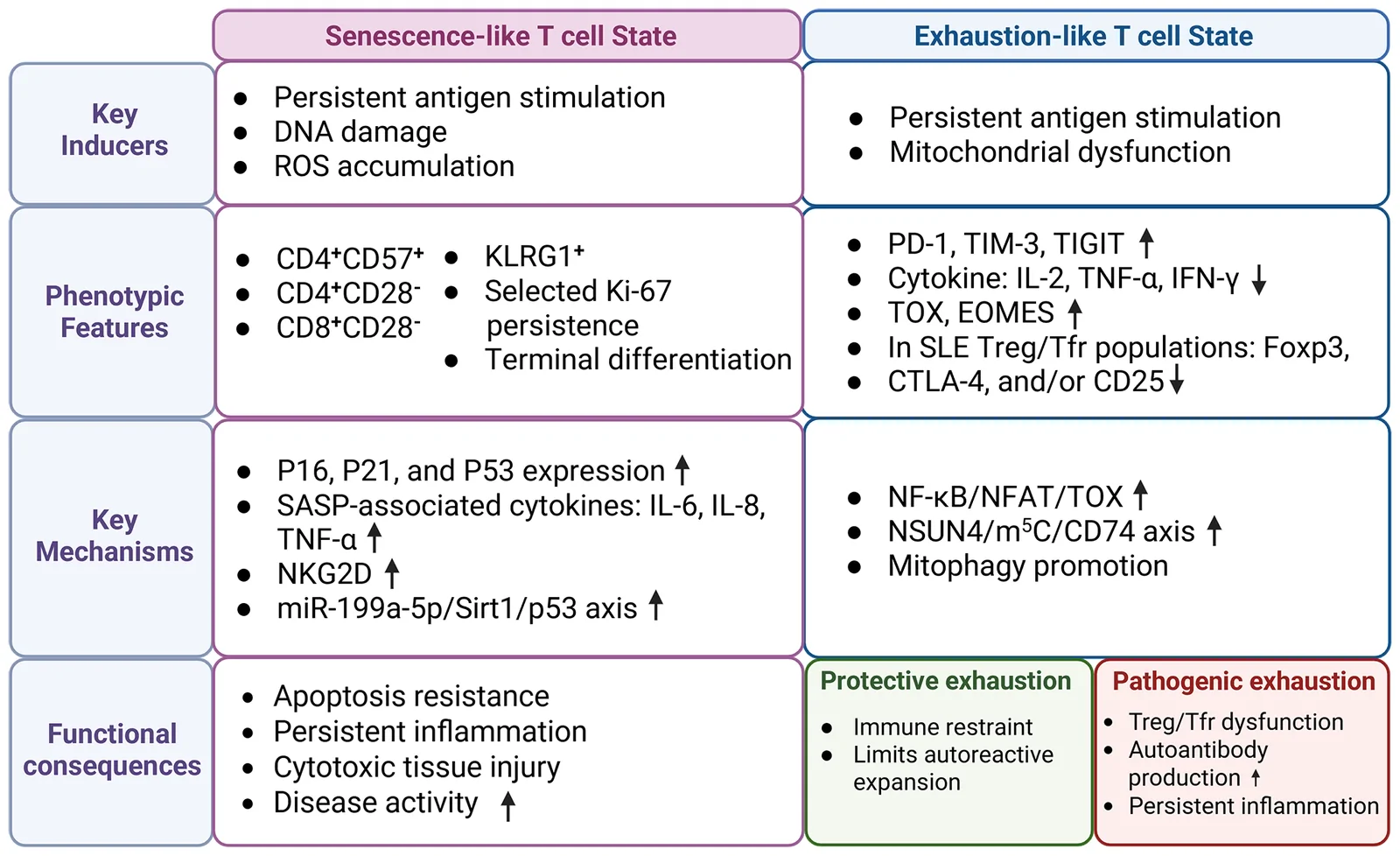
Senescent-like and exhausted-like T-cell states in SLE. Persistent antigen stimulation, DNA damage, ROS accumulation, and mitochondrial dysfunction promote senescence-like and exhaustion-like T-cell states in SLE. Senescence-like T cells are characterized by CD57 and KLRG1 upregulation, CD28 loss, terminal differentiation, and pro-inflammatory, apoptosis-resistant features. Exhaustion-like T cells show increased inhibitory receptor expression, reduced effector cytokine production, and context-dependent effects ranging from immune restraint to Treg/Tfr dysfunction, autoantibody production, and persistent inflammation. Upward and downward arrows indicate increased and decreased levels or activity, respectively. KLRG1, killer cell lectin-like receptor G1; B3GAT1, beta-1,3-glucuronyltransferase 1; SASP, senescence-associated secretory phenotype; TNF-α, tumor necrosis factor α; NKG2D, natural killer group 2 member D; miR-199a-5p, microRNA-199a-5p; SIRT1, sirtuin 1; PD-1, programmed cell death protein 1; TIM-3, T-cell immunoglobulin and mucin domain-containing protein 3; TIGIT, T-cell immunoreceptor with immunoglobulin and immunoreceptor tyrosine-based inhibitory motif domains; TOX, thymocyte selection-associated high mobility group box protein; EOMES, eomesodermin; FOXP3, forkhead box P3; CTLA-4, cytotoxic T-lymphocyte-associated protein 4; NF-κB, nuclear factor κB; NFAT, nuclear factor of activated T cells; NSUN4, NOP2/Sun RNA methyltransferase 4; m^5^C, 5-methylcytosine; CD74, major histocompatibility complex class II invariant chain; mTOR, mechanistic target of rapamycin.

## 3 Senescent-like T cells: terminal differentiation, apoptosis resistance, and pro-inflammatory potential

Cellular senescence is generally recognized as a physiological program involved in growth and development, tissue homeostasis and repair (56, 57). In contrast, CD28^-^, CD57^+^, or KLRG1^+^ T cells in peripheral blood, which are frequently referred to as “senescent T cells,” mainly reflect a terminally differentiated state induced by prolonged antigenic stimulation and are not necessarily equivalent to classically defined cellular senescence, since they may preserve potent effector functions and can be implicated in autoimmune responses (58).

Previous evidence indicates that in CD4^+^ T cells, DNA damage and elevated ROS levels trigger the AMP-activated protein kinase/TAK1-binding protein1-p38 pathway to promote a senescent-like phenotype (54). In lupus, a pioneering study first identified that the frequency of CD4^+^ CD57^+^ T cells is significantly elevated, accompanied by marked upregulation of senescence-associated biomarkers (including p16/p21/p53 mRNA, and CD57) as well as senescence-associated secretory phenotype-related cytokines such as IL-6/IL-8, and tumor necrosis factor-alpha (59). Crucially, CD4^+^ CD28^-^ T cells emerge as another prominent phenotype of senescent-like subsets (60). A clinical correlation study revealed that the elevated serum IL-15 levels in patients with SLE selectively fuel the proliferation and activation of this subset, consequently promoting pro-inflammatory cytokines secretion, and upregulating natural killer group 2, member D expression, thereby licensing its cytotoxicity (61). Compelling evidence further indicates that CD4^+^ CD28^-^ T cells accumulate substantially in patients with moderate SLE activity. Intriguingly, these cells retained proliferation marker Ki67 expression (62), highlighting that CD28 loss and other senescence-associated markers do not necessarily indicate irreversible cell-cycle arrest and supporting the use of the term “senescent-like” rather than “senescent”.

Similar immunological alterations have also been reported in CD8^+^ T cells. Clinical studies involving patients with lupus nephritis revealed a marked elevation in the absolute number of CD3^+^ CD8^+^ CD28^-^ T cells compared with healthy controls, in addition, forkhead box protein P3 (Foxp3) expression was undetectable in this subset (63, 64). Taken together with previous findings, these observations suggest that CD8^+^ CD28^-^ T cells appear to be preferentially associated with pro-inflammatory responses, particularly IFN-γ secretion (65), rather than with classical immunoregulatory function (63). Notably, CD8^+^ CD28^-^/CD57^+^ T cells may exhibit both regulatory and cytotoxic functions depending on the disease stage or the conditions (66, 67). Their functional identity should be assessed comprehensively by considering Foxp3 expression, cytotoxic effector molecules, and inflammatory cytokine profiles.

It should be further emphasized that the functional implications of T-cell senescence in SLE are not universally pathogenic. Specifically, human umbilical cord-derived mesenchymal stem cells (hUC-MSCs) have been shown to alleviate lupus-like manifestations. Mechanistically, Cheng et al. demonstrated that hUC-MSCs facilitate the senescence of splenic CD4^+^ T cells in MRL/*lpr* mice via the miR-199a-5p/sirtuin 1 (Sirt1)/p53 axis; this cascade notably downregulates Sirt1, thereby promoting p53 acetylation and the subsequent expression of p21 and p16 (68). In stark contrast to the pro-inflammatory CD4^+^ CD57^+^ T-cell expansion driven by IL-15, this hUC-MSC-induced, p53/p21/p16-mediated accelerated senescence appears to reinstate the cell-cycle checkpoint (68). Taken as a whole (**Figure 2**), senescent-like T cells in SLE exhibit profound functional plasticity, representing a terminally differentiated state orchestrated by the convergence of chronic antigenic stimulation, metabolic aberrations, and cytokine signaling. Given this functional divergence, these senescent-like populations serve as a critical cellular bridge linking metabolic reprogramming to SLE disease activity.

## 4 Exhausted-like T cells: the dual facets of protective tolerance and pathogenic loss-of-function

Unlike senescent-like T cells, exhausted-like T cells primarily reflect a state of progressive functional impairment following protracted antigen exposure (69). They typically exhibit sustained upregulation of inhibitory receptors, including PD-1, T-cell immunoglobulin and mucin-domain containing-3 (TIM-3) and T-cell immunoreceptor with immunoglobulin and ITIM domains (TIGIT), along with reduced cytokine-producing capacity (including IL-2 and IFN-γ), and blunted proliferation and cytotoxic functions. These changes are accompanied by specific transcriptional programs orchestrated by transcription factors such as thymocyte selection-associated high mobility group box protein (TOX) and eomesodermin (EOMES) (70–73). In tumor immunology, mitochondrial dysfunction is a recognized driver of exhausted-like T cells (74, 75), in which mitochondrial ROS accumulation is linked to sustained nuclear factor of activated T-cells activation and increased TOX expression (74). It should be emphasized, however, that T-cell exhaustion cannot be defined solely by PD-1 expression. T-cell exhaustion should be comprehensively evaluated using a combination of multiple inhibitory receptors, transcriptional and cytokine profiles, effector capacity, and metabolic states.

Prior research comparing T-cell transcriptomics across various autoimmune diseases and chronic infections suggested that the exhausted trait of CD8^+^ T cells in autoimmune diseases correlates with a lower relapse risk and a better prognosis (76). Consistent with this concept, Lima et al. observed that during long-term remission in patients with SLE, the proportions of CD4^+^ T-bet^+^ CD45RO^+^, CD4^+^ EOMES^+^, and CD8^+^ EOMES^+^ T cells were significantly higher than those in patients with active disease. Intriguingly, the expression levels of PD-1 and EOMES remained elevated compared with those in healthy controls, regardless of disease activity (77). Based on these observations, these findings raise the possibility that EOMES-associated, exhausted-like T cells may contribute to an active tolerogenic mechanism that sustains SLE remission.

In patients with active or severe SLE, the exhausted phenotype is intimately coupled with a loss of immunoregulatory function and aberrant T-B cell crosstalk. A single-cell transcriptomic and ATAC-seq analysis identified a distinct subset of CCR7^lo^ CD74^hi^ CD4^+^ Tregs in patients with severe SLE. Driven by IFN-I, this subset exhibited robust expression of multiple exhaustion markers (PD-1, TIM3, TIGIT, and TOX), together with an enhanced secretory capacity for pro-inflammatory cytokines, suggesting impaired regulatory function together with the acquisition of an inflammatory transcriptional state (78). Moreover, PD-1 expression on Tfr cells from patients with SLE exhibited a strong positive correlation with both anti-DNA antibody titers and disease activity, while their suppressive capacity toward Tfh cells was significantly compromised, concomitant with Foxp3, and CD25 downregulation (79). At the follicular microenvironment level, this suggests a scenario where Tfr exhaustion drives a loss of inhibitory control over Tfh-mediated B-cell activation, ultimately fueling autoimmunity (**Figure 2**).

Recently, research on CD7^hi^ CD74^hi^ CD8^+^ T cells has linked cellular metabolism with the exhausted phenotype. Mechanistically, NOP2/sun RNA methyltrasferase family member 4 maintains CD74 expression via m^5^C methylation, and promotes CD8^+^ T-cell exhaustion through the CD44/mTOR-related mitophagy pathway and ultimately exacerbates autoimmune dysregulation in SLE (**Figure 2**) (80). These insights offer a novel paradigm: exhausted-like CD8^+^ T cells are defined not merely by altered surface inhibitory receptor expression, but also by profound metabolic imbalances governed by RNA epigenetic modifications, aberrant mTOR signaling, and disrupted mitochondrial quality control. In summary, exhausted-like T cells reshape T-cell fate and function, ultimately contributing to the maintenance of inflammation, immune dysregulation, and autoantibody responses in SLE.

Collectively, senescent-like and exhausted-like T-cell states reflect heterogeneous adaptations to chronic inflammatory stimulation, rather than fixed protective or proinflammatory phenotypes. Metabolic therapies may have narrow therapeutic windows because the same pathways support protective immunity and immune tolerance, and their effects may vary among cell types and disease stages. Metabolic plasticity may also allow cells to compensate through alternative pathways. In lupus-prone mice, T-cell-specific glutaminolysis inhibition attenuated autoimmunity but impaired T-dependent humoral and antiviral Tfh-cell responses (44). Although most evidence remains preclinical, mTOR inhibition with sirolimus has shown clinical benefit in patients with SLE (81).

## 5 Conclusion

Metabolic reprogramming in T cells underpins the aberrant immune responses characteristic of SLE. Collectively, these pathways converge on three major axes: glycolytic signaling through CaMK4 and AKT/mTOR; glutamine–mitochondrial redox metabolism involving GLS, GSH, ROS, and OXPHOS; and lipid metabolism. To better understand SLE pathogenesis, future studies should characterize the dynamic changes in senescent-like and exhausted-like T cells during disease onset, active disease, remission, relapse, and the development of irreversible organ damage. Future SLE treatment strategies should focus on specific T-cell metabolic preference, driving a paradigm shift from conventional immunosuppression toward precision immunomodulation tailored to cell states and disease stages.
